# Substrate Stiffness Modulates Hypertrophic Chondrocyte Reversion and Chondrogenic Phenotype Restoration

**DOI:** 10.3390/cells14161291

**Published:** 2025-08-20

**Authors:** Da-Long Dong, Guang-Zhen Jin

**Affiliations:** 1 Institute of Tissue Regeneration Engineering (ITREN), Dankook University, Cheonan 31116, Republic of Korea; dongdalong@dankook.ac.kr; 2 Department of Nanobiomedical Science and BK21 PLUS NBM Global Research, Center for Regenerative Medicine, Dankook University, Cheonan 31116, Republic of Korea; 3 Department of Biomaterials Science, College of Dentistry, Dankook University, Cheonan 31116, Republic of Korea

**Keywords:** cartilage regeneration, chondrocyte hypertrophy, PDMS, YAP signaling, mechanical microenvironment, phenotype reversion, matrix stiffness, Smad signaling, tissue engineering

## Abstract

The stiffness of the extracellular matrix (ECM) plays a pivotal role in the progression of osteoarthritis (OA), particularly by promoting hypertrophic differentiation of chondrocytes, which hinders cartilage regeneration and accelerates pathological ossification. This study aimed to investigate how substrate stiffness modulates hypertrophic chondrocyte behavior and whether it can reverse their phenotype towards a more stable, chondrogenic state. A series of tunable polydimethylsiloxane (PDMS) substrates with stiffnesses ranging from 78 to 508 kPa were fabricated to simulate varying mechanical microenvironments. Hypertrophic chondrocytes were cultured on these substrates, and their morphology, nuclear architecture, gene/protein expression, and mechanotransductive signaling pathways were systematically evaluated. After 7 to 21 days of culture, the chondrocytes on stiffer matrices exhibited enlarged nuclei, increased cytoskeletal tension, and enhanced focal adhesion signaling. This corresponded with the upregulation of osteogenic and hypertrophic markers such as *RUNX2*, *COL10A1*, and *COL1A1*. In contrast, cells on softer substrates (78 kPa) displayed reduced nuclear YAP localization, higher levels of phosphorylated YAP, and significantly increased expression of *COL2A1* and *SOX9*, indicating reversion to a chondrogenic phenotype. Furthermore, differential activation of Smad1/5/8 and Smad2/3 pathways was observed depending on matrix stiffness, contributing to the phenotype shift. Matrix stiffness exerts a significant regulatory effect on hypertrophic chondrocytes via YAP-mediated mechanotransduction. Soft substrates promote phenotype reversion and cartilage-specific gene expression, offering a promising biomechanical strategy for cartilage tissue engineering and OA intervention.

## 1. Introduction

Osteoarthritis (OA) is a common, multifactorial degenerative joint disease marked by progressive cartilage degradation and chondrocyte dysfunction. While mechanical stress and aging are known contributors, increasing evidence highlights that biochemical and molecular factors also play essential roles in cartilage deterioration. Notably, the upregulation of matrix metalloproteinases (MMPs) and ADAMTS enzymes has been shown to accelerate matrix breakdown and cartilage degeneration [1,2]. Among the pathological processes, the phenotypic transformation of chondrocytes is particularly critical. As the only cell type in articular cartilage, any dysfunction in chondrocytes directly impacts tissue homeostasis. Studies have demonstrated that abnormalities such as apoptosis, autophagy defects, and hypertrophic differentiation are closely associated with OA progression [3,4]. Hypertrophic chondrocytes—characterized by the expression of *COL10A1, RUNX2*, and MMP13—mimic the terminal differentiation state of growth plate chondrocytes and promote endochondral ossification. Such premature hypertrophy disrupts normal cartilage repair and is frequently observed in both clinical and experimental OA samples [5,6,7,8,9,10,11,12]. In addition, inflammatory responses driven by chronic low-grade inflammation also contribute to cartilage breakdown and bone remodeling through cytokine and chemokine production [13,14]. The induction of chondrocyte hypertrophy is influenced by multiple factors, including mechanical stimuli, receptor signaling, and intracellular pathways [15,16]. As hypertrophy progresses, the expression of Type *I and X collagen,* MMP13, and other ossification-related markers increases, further aggravating the disease process [17,18]. Various signaling pathways, particularly Wnt/β-catenin and Ihh, have been implicated in regulating this transition [5,19].

Despite emerging regenerative therapies such as autologous chondrocyte implantation (ACI) and low-intensity pulsed ultrasound (LIPUS), current interventions remain insufficient to reverse chondrocyte hypertrophy or restore the native phenotype [20,21]. Therefore, elucidating the mechanisms underlying hypertrophic chondrocyte differentiation and developing effective strategies for phenotype modulation remain urgent challenges in cartilage tissue engineering [7]. Recent studies highlight the role of the mechanical microenvironment, especially ECM stiffness, in regulating chondrocyte fate. Mechanotransduction occurs primarily through Integrinβ1, focal adhesion kinase (FAK), and the YAP/TAZ signaling axis [22,23]. YAP, a key mechanosensitive transcriptional co-activator, responds to matrix elasticity by shifting its localization between the nucleus and cytoplasm, thereby modulating gene expression patterns [24,25]. Its activity is tightly linked to the RhoA/ROCK cytoskeletal tension and FAK-dependent pathways [26,27]. These factors have also been shown to regulate stem cell differentiation, proliferation, and tissue regeneration potential [28,29,30].

However, how matrix stiffness modulates hypertrophic chondrocytes specifically—and whether such mechanical cues can reverse their phenotype—remains largely unexplored. In this study, we constructed a series of PDMS-based culture platforms with controlled stiffness to simulate diverse mechanical conditions. We investigated how these mechanical environments influence chondrocyte morphology, gene/protein expression, and YAP-related signal transduction, aiming to provide novel insights into cartilage regeneration via biomechanical regulation.

## 2. Experimental Methods

### 2.1. Steps for Preparing PDMS Substrates

For the construction of PDMS substrates with different stiffnesses, Sylgard 184 and Sylgard 527 elastomer kits (Dow Corning) were mixed from 10 kPa to 508 kPa. The mixture was thoroughly stirred according to the ratio of the curing agent and degassed before finally being fully cured at room temperature for 72 h until fully crosslinked. Afterward, the cured PDMS substrates were subjected to an oxygen plasma system treatment for 5 min (CUTE, Femto-Science Inc., Hwaseong, Republic of Korea). Then, they were quickly immersed in a solution containing 10% (*v*/*v*) 3-aminopropyl triethoxysilane (APTES, Sigma-Aldrich, St. Louis, MO, USA) in 99% anhydrous ethanol and heated at 60 °C for 3 h to provide the surface of the substrate with amino functional groups. They were washed 3–5 times with sterile DW to clean the APTES. Next, the sample substrates were placed in a 2.5% (*v*/*v*) glutaraldehyde solution provided by Sigma-Aldrich, left standing for 1 h at room temperature, and washed 3–5 times with sterile PBS and rapidly sterilized with 75% alcohol. PBS was used again to rinse off residual ethanol. Finally, the substrate underwent a 2 h incubation at 37 °C in a 0.2% (*w*/*v*) type I collagen solution to finalize the coating for later application.

### 2.2. Primary Chondrocyte Culture

SD rats, aged 4 weeks, were acquired from the institution’s animal experiment center. The costal cartilage tissue was collected under aseptic conditions. Then, excess non-chondral tissues such as perichondrium, fat, and blood vessels around the chondral surface were removed with forceps under a dissection microscope, cut into small pieces of about 1 mm^3^ to facilitate enzymes, added to pre-prepared 2% collagenase type II solution, and placed, gently shaken, or knocked by hand into a 37 °C water bath for 30 min to digest. We then filtered out the undigested debris after digestion with a 200 mesh cell strainer, collected the suspension containing cells, combined it with high-glucose DMEM medium containing 10% FBS, then spun it at 2000 rpm for 3 min. We then discarded the supernatant, suspended the cells in complete DMEM medium with 10% FBS and 1% penicillin streptomycin (PS), seeded this in 100 mm culture dishes, and cultured it in a 5% CO_2_ incubator at 37 °C. The cells that were approximately 90% confluent were digested with 0.25% trypsin EDTA and passaged at a 1:3 ratio, with P4 chondrocytes being used for the subsequent experiment.

### 2.3. Cell Compatibility Test on PDMS Surfaces

A Viability/Cytotoxicity Kit for Mammalian Cells by live/dead^®^ (Cat. No. R37601, Life technologies, Carlsbad, CA, USA) was used to detect chondrocyte viability 24 h after the chondrocytes were seeded on PDMS surfaces, according to the manufacture’s instruction. Four-well plates were filled with PDMS substrates, then we added the prepared staining solution (calcein AM and ethidium homodimer-1). We incubated the samples at room temperature in dark conditions for 15 min. After that, we gently washed the samples with sterile PBS. We then observed and imaged the samples under a fluorescent microscope (FITC channel and Texas red channel).

### 2.4. SA-β-Galactosidase Staining

Cellular aging was marked by the activity of β-galactosidase (SA-β-Gal). Senescent β-gal test reagents, including SA-β-Gal working solution, staining fixative, and Safranin B, were purchased from Senescence beta galactosidase Staining Kit (G1580, Solarbio, Beijing, China). About 15 min before testing, we first fixed the cells with staining fixative, then added the SA-β-Gal working solution, and incubated the cells in dark conditions at 37 °C for 10 h. Then we observed the images using an inverted optical microscope. Green/blue green precipitates indicated positive results, while those without green/orange precipitate indicated negative results. The number of positive cells was counted using ImageJ software (version 1.53, National Institutes of Health, Bethesda, MD, USA).

### 2.5. YAP Detection via Immunofluorescence Staining

The cells were fixed with 4% PFA for 15 min after 7 days, then washed three times with PBS for 5 min each, permeabilized with 0.2% Triton X-100 for 10 min, and washed again three times in PBS for 5 min each. The cells were treated with 5% BSA for one hour. The YAP primary antibody (Santa Cruz Biotechnology, sc-101199, Dallas, TX, USA) was diluted at a 1:200 ratio in the blocking buffer and left on the cells overnight at 4 °C. The cells were washed three times in PBS for 10 min each time to remove unbound antibodies. The secondary antibody with a fluorescent label was diluted to a 1:100 ratio and incubated in the dark for one hour at room temperature. The cells had their nuclei stained with a 1 µg/mL DAPI solution for 5 to 10 min at room temperature, and were then washed three times in PBS, with each wash lasting 5 min. A confocal microscope was employed to capture images for observing YAP in the cells. Nuclei appeared blue and YAP appeared green when observed under the fluorescence microscope due to fluorescent dye Alexa Fluor 488 attached to the anti-YAP antibody.

### 2.6. COL-2 Immunofluorescence Staining

The cells were fixed with 4% paraformaldehyde for 15 min at room temperature after 21 days, then washed three times with PBS for 5 min each, treated with 0.2% Triton-X100 for 10 min to permeabilize, followed by two additional washes with PBS, each lasting 5 min. Non-specific interactions of proteins were inhibited by treating with 5% bovine serum albumin for an hour at room temperature. Overnight incubation with collagen type II primary antibody (sc-52658, Santa Cruz Biotechnology, Dallas, TX, USA) at 4 °C was performed on the cells using a 1:150 dilution in blocking buffer. The cells were washed 3 × 5 min in PBS, and non-bound primary antibodies were removed. Alexa Fluor 488, a secondary antibody with a fluorescent tag, was diluted at a ratio of 1:100 and used in the dark for an hour. A DAPI solution (1 µg/mL) was used to counter-stain the nuclei for 5–10 min. After being washed three times for five minutes in PBS, the cells were imaged with a confocal laser scanning microscope to observe where collagen type 2 interacts with the cells. Green represented the collagen II interaction site within the cells, while the nucleus appeared blue upon visualization under the fluorescence microscope after staining with DAPI dye.

### 2.7. Western Blot

Following 21 days in differentiation culture, the cells underwent PBS washing and trypsin digestion. Cells were collected after centrifugation, and the supernatant was discarded. The cells were washed again with PBS, and the cell pellets under an ice bath were lysed with lysis buffer containing protease inhibitor (Halt™ Protease and Phosphatase Inhibitor Cocktail, 100×, Thermo Scientific, Waltham, MA, USA; EBA-78440, Elpis Biotech, Daejeon, Republic of Korea) for 30 min at 4 °C on ice. The suspension was centrifuged at 10,000 rpm for 10 min at 4 °C, as instructed in the kit manual. The concentration of protein was determined with a Pierce™ BCA Protein Assay Kit (Thermo Fisher Scientific, Waltham, MA, USA). The supernatants containing the samples were heated at 100 °C for 10 min and separated by SDS-PAGE. The gel-separated samples were blotted onto PVDF membranes pre-soaked in a cold tray filled with deionized water (Bio-Rad, based in Hercules, CA, USA). The membrane was incubated with 5% SolMate BSA Grade IY (GeneAll, Seoul, South Korea) for 1 h at room temperature, then treated with primary antibodies and β-actin antibodies (as a loading control) at 4 °C overnight. The next day, the membranes were rinsed three times with TBST (15 min each time) and incubated for 1 h with HRP-conjugated anti-mouse/rabbit IgG (Cell Signaling Technology, Danvers, MA, USA). Using the LAS4000 mini (GE Healthcare Life Sciences, Uppsala, Sweden) protein imaging system by GE Healthcare, protein signals were visualized with the help of SuperSignal™ West Pico and SuperSignal™ West Pico Plus chemiluminescence detection reagents from Thermo Scientific. Images were captured with ImageJ 1.52P software (The list of antibodies used in this study is provided in the Supplementary Data Table S1).

### 2.8. Analysis Using Quantitative Real-Time PCR (qRT-PCR)

According to the manufacturer’s instructions, total RNA was isolated using TRIzol reagent from Invitrogen, located in Carlsbad, CA, USA. The synthesis of complementary DNA (cDNA) was executed with the iScript™ cDNA Synthesis Kit (Bio-Rad Laboratories, Hercules, CA, USA). Quantitative real-time PCR (qRT-PCR) amplifications were conducted using a StepOne Plus Real-Time PCR System (Applied Biosystems, Foster City, CA, USA) and SensiMix™ SYBR Hi-ROX Mix (Bioline, London, UK; Cat. No. QT-605-05). The reactions contained a total volume of 20 μL and four replicates per sample. All qPCR results were analyzed using the 2^−ΔΔCt^ method. For statistical comparisons, log-transformed 2^−ΔΔCt^ values were used in order to meet the assumption of normality required for parametric tests, such as one-way ANOVA. Prior to analysis, the normality of the log-transformed data was assessed using the Shapiro–Wilk test.

### 2.9. Alizarin Red S Staining and Quantification of Mineralization

AR staining was performed to observe the mineralization potential induced by hypertrophic differentiation of chondrocytes growing on PDMS substrates with different stiffness levels. Briefly, after a certain culture time, we poured off the culture medium and the cells underwent two washes in PBS before being fixed in 4% formaldehyde for 20 min at room temperature. After three washes with deionized water, we stained the cells with 2% AR solution (pH 4.1) for 20 min at room temperature. We washed the cells sufficiently until background removal using deionized water and air-dried them overnight at room temperature. Representative pictures were captured under an inverted optical microscope to observe the calcified deposits. To count the mineralized area, we washed the stained samples five to six times with PBS to reduce the background stain and gently blotted them dry with absorbent paper without tearing the cell layer. Fluorescence images were obtained from a fluorescence microscope equipped with a FITC channel and Texas red channel. ImageJ 1.52P was used to count mineralized areas, and statistical analyses were performed using GraphPad Prism.

### 2.10. Safranin O Staining and Quantitative Analysis

To determine the extracellular matrix synthesized during differentiation, as well as the differentiation state and maturation of the matrix produced by chondrocytes cultured under varied substrate stiffness conditions, the Safranin O staining method was used to detect GAG deposits. After 21 days of culture differentiation, the cells were rinsed twice with PBS and fixed in 4% paraformaldehyde at room temperature for 10–15 min. We covered the cells with a drop of 0.1% Safranin O solution and allowed it to incubate for 5–10 min at room temperature. When there was a need to increase contrast when the stain samples had greater amounts of GAG content, we could increase staining duration up to 15 min. The stainable samples were thoroughly washed by gentle rinsing using distilled water or PBS 2–3 times. To perform this carefully and avoid dislodging cells, a brief destaining step using 70% ethanol for 30 s–1 min was conducted on the stainable samples. After removing excessive dye by blotting gently with absorbent paper without tearing the cell layer, the stainable samples were observed under a microscope. Samples presenting with a red color indicated a GAG-rich area where a cartilage-like specific matrix was produced. Pictures were taken under a fluorescent microscope and ImageJ 1.52P software was applied for measuring the areas stained. Obtaining the results of the statistical analysis and graphical presentation was also performed using GraphPad Prism.

### 2.11. Analysis of Statistics

GraphPad Prism 8.0.2 software was used for statistical analysis, and the data were presented as mean ± SD. The differences among the groups were assessed using one-way ANOVA, followed by multiple comparison tests and an unpaired T test. Western blot or immunostaining images were quantitatively analyzed using ImageJ 1.52P software, and *p* < 0.05 was considered significant.

## 3. Result

### 3.1. Construction and Surface Characterization of PDMS Substrates

**Figure 1:**
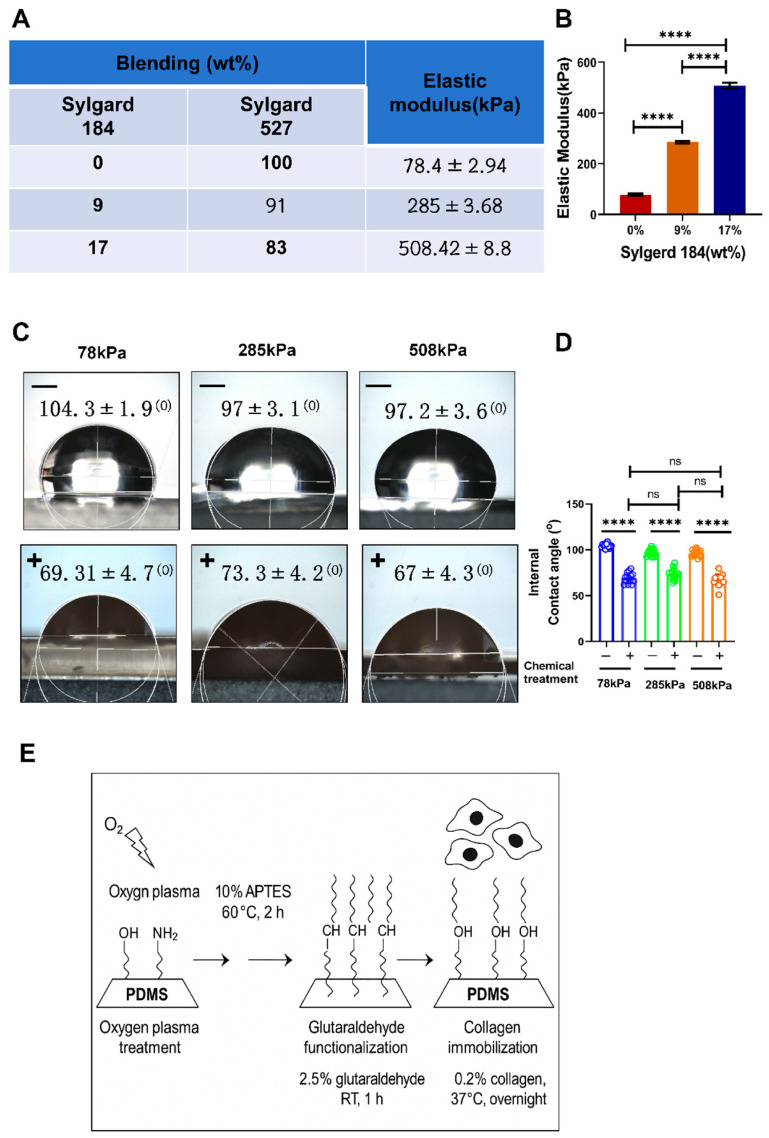
Fabrication of PDMS substrates with different stiffness levels and analysis of surface hydrophilicity modification. (**A**) Schematic illustration of PDMS substrate preparation. (**B**) Young’s modulus measurements of PDMS substrates prepared under different mixing ratios and curing conditions. The results show that the elastic modulus of the PDMS substrates can be stably maintained at approximately 78 kPa, 216/258 kPa, and 508 kPa, meeting the requirements for varying stiffness levels. (**C**) Schematic and static water contact angle measurement showing surface hydrophilicity improvement. The images display water droplet morphology on untreated (“–”) and plasma-activated type-II-collagen-coated (“+”) PDMS surfaces. (**D**) Quantitative analysis of contact angles before and after surface treatment across PDMS substrates of different stiffness levels. (**E**) Schematic diagram of the PDMS surface treatment process. Data are presented as mean ± SD, **** *p* < 0.0001, ns: not significant.

To determine whether substrate stiffness regulated the hypertrophic phenotype of chondrocytes, we first prepared three kinds of PDMS with different stiff substrates: 78 kPa, 258 kPa, and 508 kPa (Figure 1A). We found that the Young’s modulus of PDMS could be stably controlled around 78 kPa, 258 kPa, and 508 kPa by changing the curing temperature and the proportion of prepolymer and curer during mixing (*n* = 6/group), which can meet the mechanical requirements of cell culture (Figure 1B). On this basis, in order to better attach cells on the PDMS surface, we further carried out hydrophilicity enhancement treatment. The results of the static water contact angle show that, before treatment, the “–” (without treating) group has obvious hydrophobicity, and its static water contact angle exceeds 90°; moreover, after plasma activation and collagen coating, the “+” (treated) group has an extremely small contact angle (<30°), indicating a good improvement effect on surface hydrophilicity. Quantitative results also show that all three stiffness groups exhibit enhanced hydrophilicity after being treated (Figure 1C,D). Figure 1E shows the modification process of PDMS surfaces: First, activate the surface of PDMS by plasma; then, use collagen to coat type II collagen, so as to achieve the purpose of enhancing the hydrophilicity of PDMS and promoting cell attachment, thus solving the inherent hydrophobicity defect of PDMS under the condition of reducing cell adhesion and differentiation. In summary, the PDMS substrates constructed have adjustable stiffness and enhance hydrophilicity at their surfaces. Such stiff PDMS substrates will provide favorable physical conditions for us to study the phenotypic changes in hypertrophic chondrocytes in various situations.

### 3.2. Impact of PDMS Substrate Rigidity on Aging and Survival of Enlarged Chondrocytes

**Figure 2:**
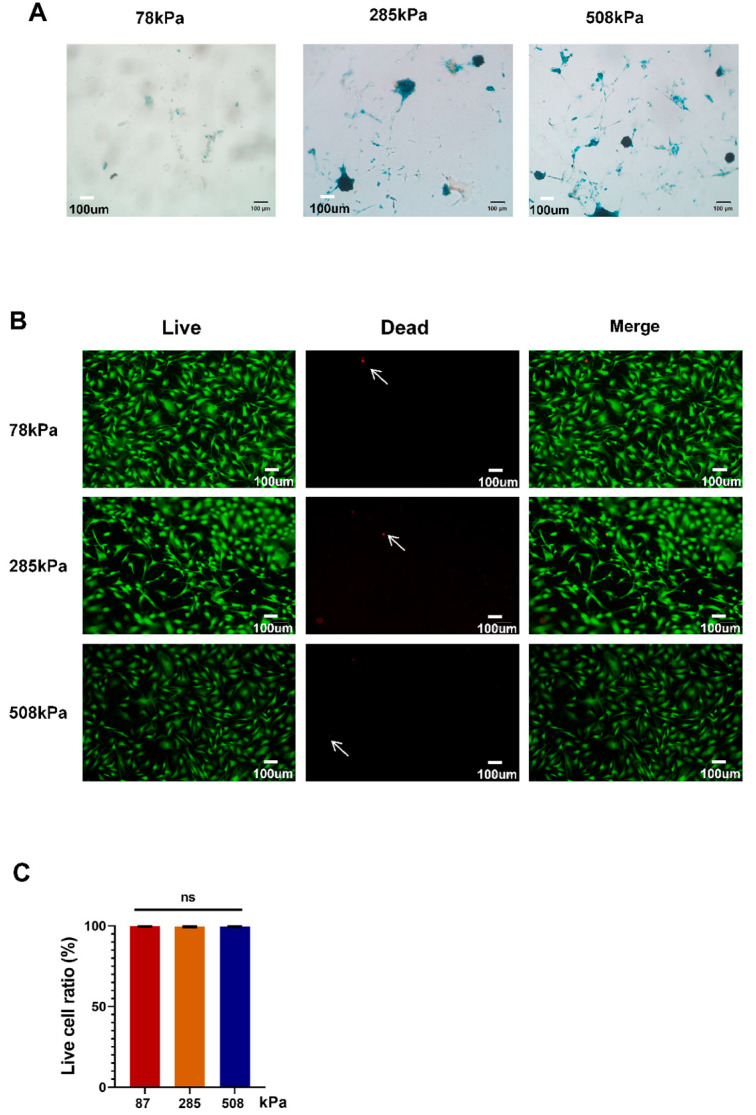
Impact of PDMS substrate rigidity on the aging and survival of hypertrophic chondrocytes. (**A**) SA-β-Gal staining of hypertrophic chondrocytes cultured on PDMS substrates with different stiffness levels. (**B**) Live/dead staining of cells cultured on PDMS substrates of varying stiffness. Green (Calcein-AM) labels live cells, while red (PI) marks dead cells. Arrows indicate PI-positive dead cells. (**C**) Quantification of live cell ratio. Data are presented as mean ± SD, ns: not significant.

Next, we further explored how PDMS substrate stiffness affects cellular senescence/viability of hypertrophic chondrocytes by performing SA-beta-Gal staining and live/dead cell assays on 78 kPa-, 285 kPa-, and 508 kPa elastic modulus substrates. Figure 2A shows more blue-colored positive areas after being stained with SA-beta-Gal in higher stiffness samples, indicating that increasing the stiffness level induces hypertrophic chondrocyte cells to age more easily. Observing carefully, we find there are more positive cells from cultures grown at stiffnesses of 285 kPa and 508 kPa than 78 kPa. This result suggests that substrate stiffness upregulates cellular senescence of hypertrophic chondrocytes. Double labeling with Calcein AM/PI proved that cell viabilities remain unchanged after being exposed to different substrate stiffness levels; moreover, bright-field (Figure 2B) and quantitative pictures present as clearly green in color in every group, with several scattered red ones along it (Figure 2B,C), respectively. The numbers of dead/hanging cells displayed using quantifying results shown in Figure 2C suggest that those grown at these three stiffnesses have survival ratios above 95%; therefore, the viability of hypochondrocytes grown under all three elastic moduli is quite high. Conclusively, although PDMS stiff substrates do not significantly affect cell viability of hypertrophic chondrocytes, substrate stiffness causes mild yet significant cellular senescence of chondrocytes.

### 3.3. Increased Substrate Stiffness Induces Nuclear Enlargement in Hypertrophic Chondrocytes

**Figure 3:**
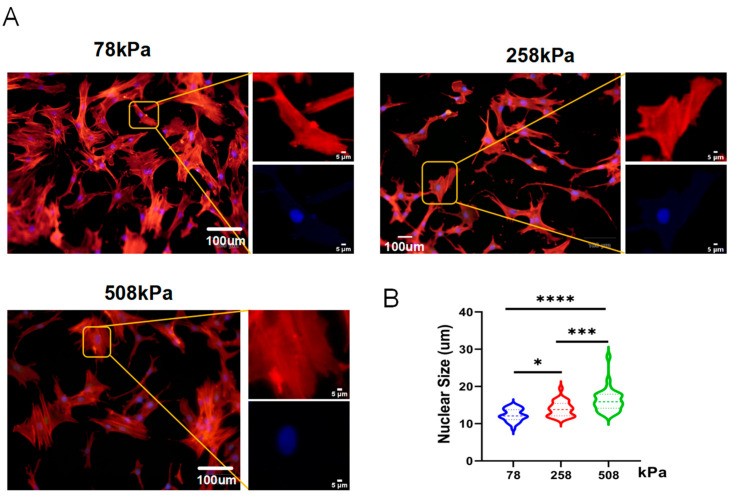
Nuclear morphology of hypertrophic chondrocytes is modulated by substrate stiffness. (**A**) Representative immunofluorescence images of hypertrophic chondrocytes cultured on PDMS substrates of different stiffness levels (78, 258, and 508 kPa) for 21 days. F-actin (red) and nuclei (DAPI, blue) are shown. Insets highlight nuclear morphology. (**B**) Quantification of nuclear size from image analysis, revealing a significant increase in nuclear area with increasing substrate stiffness. (*n* = 30). The data are shown as mean ± SD, * *p* < 0.05, *** *p* < 0.001, **** *p* < 0.0001.

Chondrocyte hypertrophy is characterized by a drastic change in cell size and volume, which greatly influences the nucleus size. This study revealed that hypertrophic chondrocytes can increase its volume by over 500% through cytoplasmic and nuclear swelling rather than organelle expansion [12]. During the process of hypertrophy, the inhibition of some membrane transporters induced fluid retention to promote cell volumetric growth [31]. Volumetric growth promotes differentiation toward osteoblasts, which are important for endochondral ossification [32], whereas nuclear enlargement enables chromatin rearrangement and gene regulation during hypertrophy [33,34]. However, excessive hypertrophy has been associated with pathological diseases [12]. In order to elucidate how matrix stiffness influenced the morphology of hypertrophic chondrocytes, we monitored changes in nuclear shape after 7 days on the PDMS substrates at stiffness values of 78 kPa, 258 kPa, and 508 kPa (Figure 3A). The immunofluorescence staining results showed that nuclei became larger as matrix stiffness increased. Quantified data (Figure 3B) showed that cells grown on the softest surface (78 kPa) had smaller nuclei than those cultured on 258 kPa and 508 kPa; however, there was no difference between 258 kPa and 508 kPa. Thus, our observations suggest that the matrix-induced change in nuclear morphology occurs through alteration in intracellular tension generated by cytoskeletal-tension-mediated mechanotransduction because the stiffer substratum would result in stronger actomyosin contractility. This results in enhanced intracellular tension transduced into the nucleus through LINC complexes to deform it, and such mechanical signaling has also been reported to induce chromatin rearrangement and activate transcriptional programs involved in osteogenesis. Therefore, the large-sized nuclei on stiff substrata should reflect hypertrophic chondrocytes undergoing an osteogenic phenotypic shift and deviating away from a chondrogenic one. Contrarywise, the small, rounder-shaped nuclei obtained on 78 kPa suggested less pressure applied to cells, revealing the native-like phenotype of chondrocytes.

### 3.4. Soft Substrates Attenuate Hypertrophic Chondrocyte Phenotype by Modulating Cytoskeletal Architecture and Focal Adhesion Signaling

**Figure 4:**
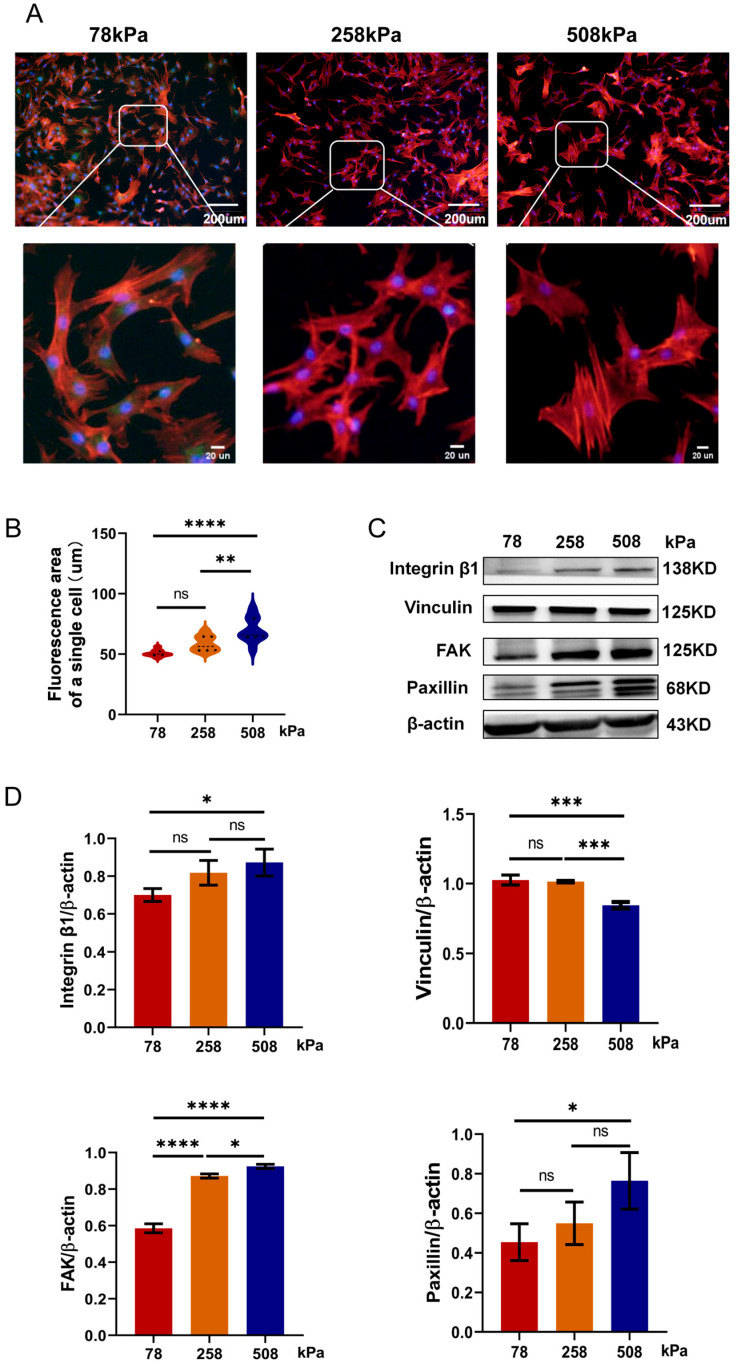
Substrate stiffness modulates cytoskeletal organization and focal adhesion protein expression in hypertrophic chondrocytes. (**A**) Representative immunofluorescence images of hypertrophic chondrocytes cultured on PDMS substrates of different stiffness levels (78 kPa, 258 kPa, and 508 kPa) for 21 days. F-actin was stained in red, while nuclei were stained in blue in the cells. Enlarged views highlight differences in cytoskeletal architecture. (**B**) Quantification of the average fluorescence area of individual cells, showing significantly increased cell spreading on stiffer substrates. (**C**) Western blot analysis of focal adhesion-related proteins, including Integrinβ1, vinculin, FAK, and paxillin in chondrocytes cultured on PDMS substrates of varying stiffness. β-actin served as a loading control. (**D**) Protein expression was quantified densitometrically and normalized against β-actin. The data are shown as mean ± SD. Statistical significance is indicated as follows: ns = not significant, * *p* < 0.05, ** *p* < 0.01, *** *p* < 0.001, **** *p* < 0.0001.

Soft substrates can regulate the phenotype of hypertrophic chondrocytes. Chondrocytes cultured on compliant matrices have undergone significant changes in cytoskeletal structures and focal adhesion signals related to switching. Cells cultured on soft substrates display a cellular morphology closer to that of native chondrocytes [35]. Moreover, the viscoelasticity of the matrix is expected to influence the mechanobiology behavior of the chondrocytes. As shown in Figure 4A, after being cultivated on different stiffness (78 kPa, 258 kPa, and 508 kPa) PDMS for 7 days, hypertrophic chondrocytes displayed great differences in cell morphology and cytoskeletal organization. With increasing stiffness, cells appeared more extended, and their cytoskeletons were stronger. These results were reflected by bigger cell sizes and clearer central arrangement stress fibers. The quantification of the single-cell fluorescence area in Figure 4B also demonstrated that cells grown on 258 kPa and 508 kPa presented significantly bigger projected areas than those grown on 78 kPa, suggesting that stiffer environments could promote cytoskeletal remodeling and stress fiber formation. At the protein level, we further identified the corresponding stiffness-dependent regulatory mechanisms from Figure 4C,D (full-length, uncropped western blots corresponding to the proteins in Figure 4 are shown in Supplementary Figure S2). To further investigate focal adhesion formation across substrates of varying stiffness, we performed immunofluorescence staining for paxillin, as shown in the Supplementary Data (Supplementary Figure S1). The proteins of Integrinβ1, vinculin, focal adhesion kinase (FAK), and paxillin related to adhesion were upregulated in the medium- and high-stiffness groups (258 kPa and 508 kPa). Specifically, there was an obvious difference between two subgroups: the FAK expression level in the 508 kPa group was much higher than that in the 78 kPa group. These results suggest that cells increase their adhesive complex and mechanosensing response when exposed to stiff environments to strengthen the cytoskeleton, resulting in persistent or aggravation of hypertrophy-related signaling pathways in this situation. In contrast, cells cultured on the softer substrate still had a relatively compact morphology and expressed fewer stress fibers, as well as lower levels of related proteins. This cellular appearance is quite similar to what was seen in healthy cartilage, which is consistent with our goal. In a low-stiffness microenvironment imitating the mechanical environment of the articular cartilage in vivo, hypertrophic chondrocytes experience significantly less mechanotransduction activity; therefore, the cell’s focal adhesion and cytoskeletal remodeling activities will be inhibited. Consequently, their hypertrophic phenotypes would eventually be suppressed, reversed, or even eliminated. In summary, our study demonstrates that tuning the stiffness of the culture substrate—especially using softer matrices such as 78 kPa—effectively inhibits the activation of hypertrophic signaling pathways, which can sustain or reverse hypertrophy-related disease conditions.

### 3.5. Substrate Stiffness Modulates YAP Subcellular Localization and Phosphorylation in Hypertrophic Chondrocytes

**Figure 5:**
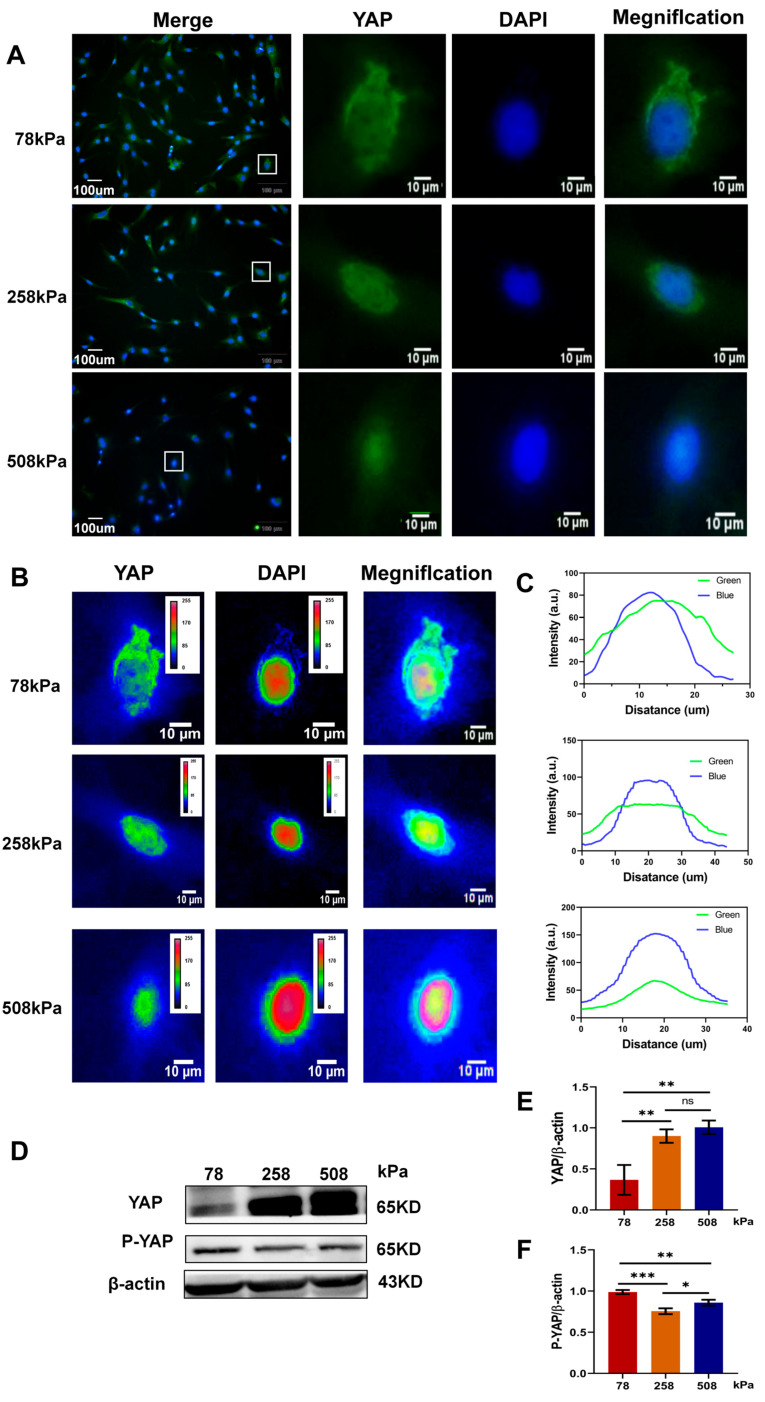
Substrate stiffness regulates YAP subcellular localization and phosphorylation in hypertrophic chondrocytes. (**A**) Representative immunofluorescence images of YAP (green) and nuclei (blue) in hypertrophic chondrocytes cultured on PDMS substrates of different stiffness levels (78 kPa, 258 kPa, and 508 kPa) for 21 days. (**B**) Heatmap representations of YAP fluorescence intensity in individual cells, revealing differential localization patterns under different substrate stiffness conditions. (**C**) Quantitative intensity profiles of YAP (green) and DAPI (blue) signals across the cell section analyzed using ImageJ, confirming the extent of YAP nuclear translocation. (**D**) Analysis of total YAP and phosphorylated YAP (P-YAP) via western blot in hypertrophic chondrocytes grown on PDMS substrates with different stiffness levels. β-actin served as a loading control. (**E**,**F**) Quantification of total YAP and P-YAP expression levels normalized to β-actin. The white box indicates the region of interest shown in the magnified view. Data represent mean ± SD, *n* = 3. Statistical significance: * *p* < 0.05, ** *p* < 0.01, *** *p* < 0.001; ns = not significant.

We speculated that the hypertrophic chondrocytes on different stiff PDMS could sense the matrix stiffness information and resulted in the stiffness-dependence translocation of YAP from cytoplasm to nucleus. We tested this hypothesis by investigating the effect of substrate stiffness on YAP localization and phosphorylation in cultured hypertrophic chondrocytes. In Figure 5A, we showed immunofluorescence staining for YAP on PDMS substrates with an elastic modulus of 78, 258, and 508 kPa, respectively. Our results show that YAP fluorescence was mainly located inside the cell body (i.e., the cytoplasm) while barely overlapped on softer 78 kPa; however, it gradually occupied a bigger nuclear area on stiffer matrices (258 and 508 kPa). The single-cell heat map displayed increased nuclear intensity on stiff matrices (Figure 5B); moreover, line-scan quantified data confirmed our observation as shown in Figure 5C, where there were increased peak nuclei at 258 and 508 kPa compared with those at 78 kPa. Western blots indicated increased total YAP expression but higher phosphorylated YAP level on the soft 78 kPa substrate than on the stiffer ones (Figure 5D,E) (full-length, uncropped western blots corresponding to the proteins in Figure 5 are shown in Supplementary Figure S3), which corresponded with densitometric analysis, revealing that the YAP/β-actin ratio was elevated at 258 and 508 kPa while the P-YAP/β-actin ratio was dramatically increased at 78 kPa (Figure 5F). These results suggested that substrate stiffness influenced YAP activation and regulated the fates of hypertrophic chondrocytes through affecting YAP localization in the nucleus and activating its downstream transcription factors. YAP remained phosphorylated and sequestered in the cytoplasmic compartment on 78 kPa, so it is possible that this phenomenon might result in suppressing or reversing the hypertrophy process. Moreover, the enhanced nuclear translocation reflects YAP mechanotransduction and promotes chondrocyte hypertrophy on stiffer substrates. Therefore, these results supported the idea that biophysical information (matrix stiffness) affected YAP signaling, which would further influence the fates of hypertrophic chondrocytes. Upregulated levels of phosphorylated YAP, together with decreased nuclear location, suggest that a compliant mechanical milieu could dampen the hypertrophic progress and help maintain the stable chondrocyte phenotype.

### 3.6. Low-Stiffness Substrate Suppresses Osteogenic Differentiation and Preserves the Chondrocyte Phenotype in Hypertrophic Chondrocytes

**Figure 6:**
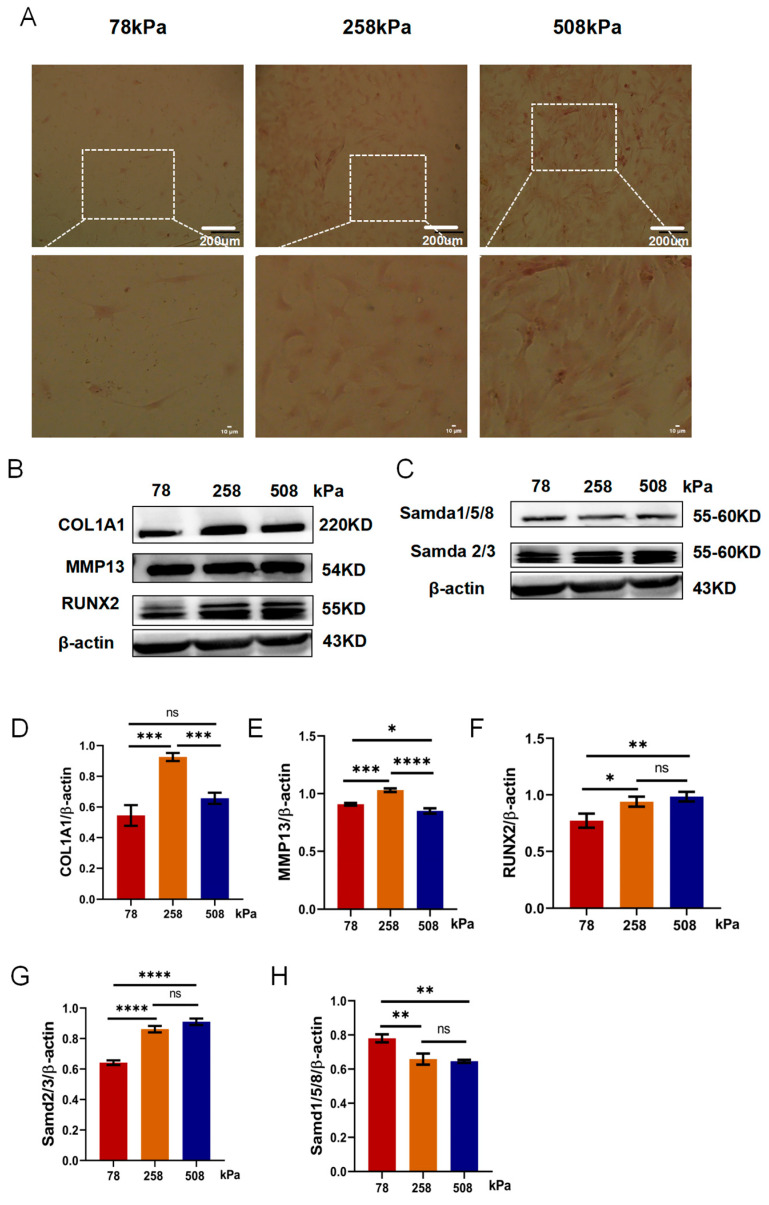
Soft substrates inhibit osteogenic differentiation and support the maintenance of chondrocyte phenotype in hypertrophic chondrocytes. (**A**) Representative images of Alizarin Red Sstaining in 24–well plates showing calcium deposition by hypertrophic chondrocytes cultured on PDMS substrates of different stiffness levels (78, 258, and 508 kPa) for 21 days. Microscopic images of Alizarin Red S staining showing matrix mineralization at higher magnification. (**B**) Western blot analysis of osteogenic and hypertrophic markers, including COL-1, MMP13, and RUNX2, across different substrate stiffness conditions. (**C**) Western blot analysis of TGF-β/BMP downstream effectors Smad2/3 and Smad1/5/8 in hypertrophic chondrocytes. (**D**–**F**) Quantification of protein levels of COL-1, MMP13, and RUNX2 normalized to β-actin. (**G**,**H**) Quantification of Smad2/3 and Smad1/5/8 protein levels. The data are shown as mean ± SD, with * *p* < 0.05, ** *p* < 0.01, *** *p* < 0.001, and **** *p* < 0.0001; ns indicates not significant.

We cultured hypertrophic chondrocytes on PDMS substrates with stiffness levels of 78, 258, and 508 kPa for 21 days to investigate the influence of stiffness on osteogenic differentiation of hypertrophic chondrocytes. After Alizarin Red S staining, we found that more calcium deposition appeared in the culture group of 508 kPa than those of 258 and 78 kPa, while the least staining was observed in the 78 kPa group (Figure 6A). Contrary results were observed in the 78kPa group, as very little mineralization occurred in the culture group of 78 kPa compared to those of 258 and 508 kPa. These data suggested that stiff substrates could induce osteogenic activity of hypertrophic chondrocytes. Then, to further verify the osteogenic ability of hypertrophic chondrocytes under different mechanical environments, we analyzed the expression of osteogenic markers by western blotting (Figure 6C–H). Type I collagen (COL-1) is an important extracellular matrix during bone formation. Its expression was lower in the 78 kPa group than it was in both the 258 and 508 kPa groups (Figure 6F). Runx2 is a master transcription factor that regulates osteogenic differentiation. It also showed the following trend: its expression was significantly lower in the 78 kPa group than it was in the two other stiffness groups (Figure 6H). Collectively, these results suggested that elevated stiffness could induce osteogenic transition of hypertrophic chondrocytes but would be inhibited by softer ones like 78 kPa. Western blots were used to measure Smad signal pathway activation, and the downstream effectors were activated by TGF-β/BMP signals (Figure 6B–H) (full-length, uncropped western blots corresponding to the proteins in Figure 6 are shown in Supplementary Figure S4.). Interestingly, the expressions of Smad2/3 were significantly lower in the 78 kPa group than they were in both the 258 and 508 kPa groups (Figure 6G); however, the expressions of Smad1/5/8 were higher in the 78 kPa group than they were in the two other stiffness groups (Figure 6H). This suggests that low substrate stiffness might preferentially maintain high levels of Smad1/5/8 signaling related to maintaining the lineage toward cartilage, not bone. Finally, the decreased mineralization reduced osteogenic markers and maintained Smad1/5/8 signaling in the 78 kPa group, suggesting that the soft environment might effectively reverse hypertrophic features and retain the chondrocyte phenotype. Therefore, these data indicated that the soft substrates attenuate the terminal osteogenic differentiation of hypertrophic chondrocytes and provided an approach to maintain chondral identity.

### 3.7. Soft Substrates Promote Redifferentiation of Hypertrophic Chondrocytes

**Figure 7:**
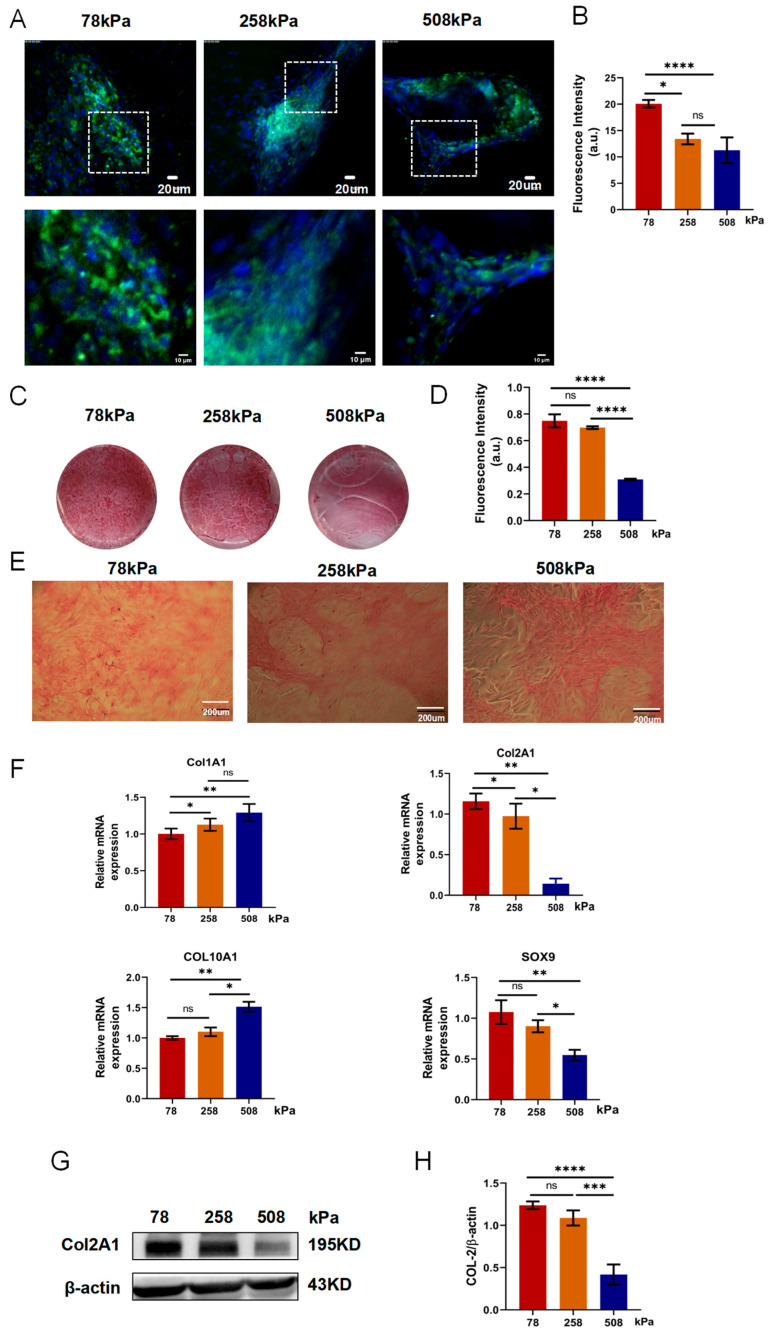
Substrate stiffness modulates chondrocyte phenotype in hypertrophic chondrocytes. (**A**) Immunofluorescent staining of type II collagen (COL2A1) in hypertrophic chondrocytes cultured for 21 days on PDMS substrates with stiffness of 78 kPa, 216 kPa, and 508 kPa. Green: COL2A1; blue: nuclei (DAPI). (**B**) Quantification of COL2A1 fluorescence intensity in different stiffness groups. (**C**) Macroscopic images of Safranin-O-stained PDMS surfaces after 21 days of culture. Red staining reflects cartilage matrix deposition. (**D**) Quantification of Safranin O staining intensity. The 508 kPa group shows significantly reduced matrix formation. (**E**) Microscopic images of Safranin O staining from (**C**), showing matrix distribution at higher magnification. Scale bars: 200 μm. (**F**) qPCR analysis of gene expression levels of Col1a1, Col2a1, Col10a1, and Sox9 in cells cultured on PDMS substrates of varying stiffness. Lower stiffness (78 kPa) maintains chondrogenic markers (Col2a1, Sox9), while higher stiffness (508 kPa) upregulates hypertrophic and fibrotic markers (Col10a1, Col1a1). (**G**) Western blot analysis of COL2A1 protein levels, normalized to β-actin, showing reduced expression on stiffer substrates. (**H**) Quantification of COL2A1 protein levels (COL2A1/β-actin ratio) from (**G**). Data are presented as mean ± SD, * *p* < 0.05, ** *p* < 0.01, *** *p* < 0.001, **** *p* < 0.0001, ns: not significant.

The factors shown in Table 1 (A1) and glycosaminoglycans (GAGs) are two fundamental components of the cartilage extracellular matrix and are widely recognized as hallmark markers of the chondrogenic phenotype. The expression levels of *COL2A1* and the deposition of GAGs reflect the biosynthetic activity of chondrocytes and are commonly used indicators for evaluating chondrogenic differentiation and matrix-forming capacity. In hypertrophic chondrocytes, sustaining high levels of *COL2A1* and GAG synthesis is critical to preserving their chondrogenic identity and preventing pathological transitions such as fibrosis or ossification [36,37,38]. PDMS substrates with stiffness levels of 78 kPa, 258 kPa, and 508 kPa were employed to study how substrate stiffness influences the phenotype of hypertrophic chondrocytes. *COL2A1* immunofluorescence staining results (Figure 7A) and statistical fluorescence intensity (Figure 7B) showed that *COL2A1* expression was highest in the 78 kPa group, slightly lower in the 216 kPa group, and obviously weakened in the 508 kPa group. Safranin O staining results confirmed the following findings (Figure 7C,E): the red-stained areas of the 78 kPa group and 258 kPa group were relatively large, and there were obvious differences between the groups; furthermore, the matrix deposited by cells in the culture plate of the 508 kPa group was poor, with a weakly red-stained area and a patchy appearance. There was no significant difference when counted (Figure 7D), but compared with the other two, the formation of the matrix in the 508 kPa group was significantly reduced. At the gene level, qPCR test data also showed that *COL2A1* and *SOX9* were expressed at the highest levels in the 78 kPa group and gradually decreased as stiffness increased, and the lowest was in the 508 kPa group (Figure 7F). The hypertrophic and fibrotic marker proteins *COL10A1* and *COL1A1* were strongly upregulated in the 508 kPa group, indicating that higher stiffness promoted the transition of the hypertrophic or fibrotic phenotype. At the protein level, western blot testing data further proved the following conclusion: the quantity of *COL2A1*′s protein expressed was highest in the 78 kPa group and lowest in the 508 kPa group, and, after normalization to β-actin, it exhibited statistical significance (Figure 7G,H) (full-length, uncropped western blots corresponding to the proteins in Figure 7 are shown in Supplementary Figure S5). The gene expression results were consistent with those obtained from western blots. In summary, softer matrices are beneficial for maintaining the hypertrophic chondrocyte’s chondrogenic phenotype, increasing the quantity of *COL2A1*, and promoting cartilage matrix synthesis. However, stiffer substrates can induce hypertrophic or fibrotic phenotype transitions, thereby suppressing their typical chondrogenic features.

### 3.8. Matrix Stiffness Regulates Chondrocyte Fate via YAP/Smad- and Integrinβ1-Mediated Signaling Pathways

**Figure 8:**
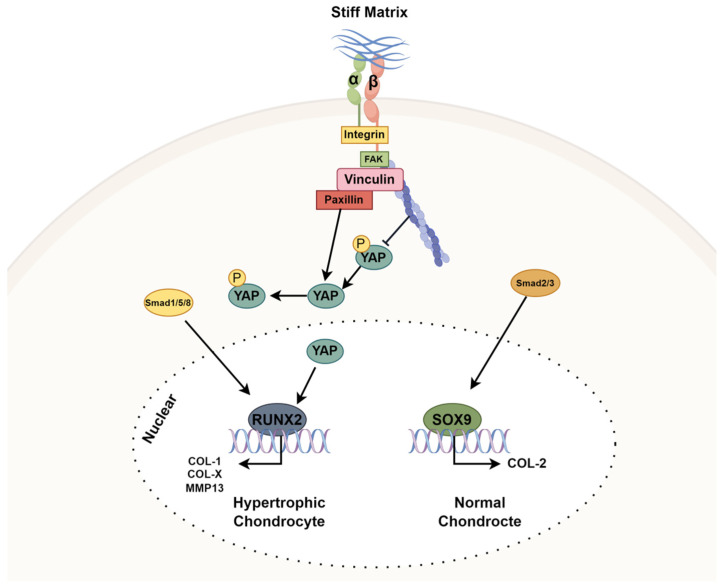
Matrix stiffness regulates chondrocyte fate via Integrinβ1–YAP and Smad signaling pathways. Schematic representation of the molecular mechanisms by which a stiff extracellular matrix (ECM) promotes chondrocyte hypertrophy. Under stiff matrix conditions, Integrinβ1 signaling activates focal adhesion kinase (FAK), paxillin, and vinculin, enhancing YAP nuclear translocation by reducing its phosphorylation. Nuclear YAP cooperates with Smad1/5/8 to induce RUNX2 expression, thereby promoting hypertrophic markers such as COL1A1, COL10A1, and MMP13. In contrast, on soft matrices, YAP remains phosphorylated and cytoplasmic, allowing Smad2/3 and SOX9 to drive COL2A1 expression, maintaining a normal chondrocyte phenotype. This figure highlights the stiffness-dependent switch between chondrogenic maintenance and hypertrophic differentiation.

To decipher the hidden mechanism of matrix stiffness controlling chondrocyte fate, we proposed a schematic model coupling mechanotransduction and transcriptional regulation (Figure 8). Under stiff matrix conditions, Integrinβ1-mediated focal adhesion signal is activated by recruiting FAK, paxillin, and vinculin. The activation of this mechanical input subsequently activates YAP translocation into the nucleus after lowering its phosphorylation level. Inside of the nucleus, active YAP promotes the expression of RUNX2, a hypertrophic determinant, via upregulating the expression of the RUNX2 gene. Therefore, YAP contributes towards promoting the expression of hypertrophy-related genes like COL1A1, COL10A1, and MMP13. In addition to YAP, Smad1/5/8 signaling also activates simultaneously with nuclear YAP for boosting the RUNX2-driven program in response to stiff substrate conditions. On a soft substrate, YAP stays mostly phosphorylated due to low nuclear entry, so it does not have a great deal of transcriptional capacity.

Cytoplasmic sequestration of YAP shifts the transcriptional balance towards the SOX9 promoter and results in the promotion of COL2A1 expression and blocking of hypertrophic transition by suppressing the SOX9-mediated program in chondrocytes. Furthermore, activated Smad2/3 also promotes SOX9-mediated gene expression on a soft substrate but not under stiff conditions. Our data revealed that the phenotype outcome is governed by an interplay between Integrinβ1-FAK-YAP and Smad signals. We conclude that PDMS substrates regulate the chondrocytic phenotypes through two different routes.

## 4. Results, Discussion, and Outlook

Based on our understanding of the relationship between extracellular matrix stiffness and chondrocyte function, we further investigated the molecular mechanisms and the regulatory roles of the signaling pathways involved. On one side, the YAP signaling controls the chondrocyte phenotype and hypertrophic differentiation process while, at another side, the stiff PDMS substrates control both processes simultaneously. Moreover, we systematically studied how varying the stiffness of PDMS substrates affected the hypertrophic status of chondrocytes and delineated the regulatory role of YAP on the hypertrophic differentiation process. It was concluded from our results that increasing the stiffness of the PDMS substrate significantly promoted the hypertrophic status of chondrocytes, while softer substrates reversed the tendency of hypertrophic differentiation and strongly elevated the expression of specific chondrocyte marker mRNA, such as COL2 and SOX9, on day 7 on the 78 kPa PDMS substrate. Chondrocytes treated on 78 kPa PDMS showed a higher cytoplasmic phosphorylated YAP level, along with highly decreased nuclear accumulation, than 258 kPa and 508 kPa, indicating the retention of YAP in cytosol by softer substrates to promote the dedifferentiation trend of hypertrophic cells, thus facilitating their maintenance of a normal chondrocyte state.

There was an ECM regulatory effect on the fate of chondrocytes. Some studies have conflicting opinions. Although most research results show that changing the physical and chemical characteristics of ECM can promote or inhibit the proliferation, differentiation, and apoptosis of chondrocytes, the regulatory mechanism is still not clear. However, some research has found that increasing the stiffness of the extracellular matrix can promote the differentiation and matrix synthesis of chondrocytes through certain ways, which is beneficial to cartilage repair and regeneration. This kind of phenomenon is mainly caused by TGF-β pathway activation, WNT pathway activation, etc., but these two kinds of pathways are essential in the chondrogenesis and maintenance of cartilage [39]. The enhanced matrix rigidity enhances the TGF-β signal activity, which upregulates genes related to chondrocyte maturation, such as SOX9 and COL2A1, which are necessary for cartilage development [40,41]. Also, the enhanced ECM rigidity increases the WNT signaling pathway involved in chondrocyte development, which also regulates proteoglycans and glycosaminoglycans required for cartilage matrix integrity, which improves cartilage strength [40,42]. In addition, a stiffer environment stimulates the proliferation of bone marrow mesenchymal stem cells (BMSCs) and promotes BMSCs’ differentiation into chondrocytes, so it is conducive to effective cartilage repair.

However, another group of researchers concluded that the excessive rigidity of ECM induces senescence and even programmed cell death in chondrocytes, promoting the onset of OA [43]. There may be differences in models, methods, and other aspects among various studies. It has been shown that softening of ECM reduces the histone deacetylase 3 (HDAC3) level of the cell membrane, causing high levels of acetylated Parkin and excessive autophagy with mitochondria, which accelerates chondrocyte senescence [8]. A stiff extracellular matrix induces Klotho gene methylation, resulting in decreased Klotho expression and accelerating chondrocyte aging [9]. In addition, mechanical stress produced by the stiff matrix leads to an increase in oxidative stress, which increases chondrocyte senescence and chondrocyte apoptosis [10]. There is an accumulation of senescent chondrocytes secreting more pro-inflammatory cytokines, accelerating cartilage degradation and inflammation—hallmark features of OA [11]. Though many researchers have confirmed that ECM rigidity plays an essential role in OA, in addition to this, there are many other things associated with OA, such as genetics, lifestyle, and inflammation, which need to be addressed effectively.

In addition, the interaction between ECM components and chondrocyte surface receptors and downstream signaling pathways is still controversial. Integrinβ1 is a well-known chondrocyte surface receptor, but controversy still exists about the roles of different Integrinβ1 types under various conditions and how they interact synergistically with each other. In addition, how other matrix molecules (such as proteoglycans and glycosaminoglycans) interact with chondrocyte surface receptors and influence cell fate is also unknown. These controversies should be resolved so that we can have a complete understanding of how ECM influences chondrocyte fate, which would provide us with a solid theoretical foundation for using the ECM as a therapeutic agent for cartilage diseases.

The above studies mainly use some basic experimental techniques to clarify the relationship between the stiffness of the extracellular matrix and OA; however, our work starts from the principles of tissue engineering, constructs an artificial microenvironment simulating the cartilage environment, and focuses on how the stiffness of the extracellular matrix regulates the dedifferentiation of hypertrophic chondrocytes through YAP signaling to facilitate clinical translation.

Many studies have shown that, during mechanotransduction, the activity of YAP depends on the rigidity of the extracellular matrix, and its translocation within cells plays a key role in this process. These results demonstrate the close relationship between YAP signaling and rigidity; therefore, regulating the rigidity of the extracellular matrix would significantly affect the activity of YAP signaling, reverse chondrocyte hypertrophy, and maintain the chondrocyte phenotype when appropriate rigidity existed.

Our data confirm the important role of YAP phosphorylation in maintaining the chondrocyte phenotype and reversing hypertrophy. Although our work demonstrates that certain ECM levels can effectively reverse hypertrophy and maintain the phenotype of chondrocytes, several points deserve further discussion. Our two-dimensional model successfully imitates the inhibitory effect of the varied ECM levels on the hypertrophic chondrocytes and the maintenance of the phenotype of the chondrocytes. However, it cannot fully substitute for the factors present in the intricate in vivo environment, including the diverse cytokines and biochemical signals that regulate the phenomenon.

We believe that, in the future, these works will deepen our understanding by performing experiments under three-dimensional conditions, further mimicking the in vivo environment to study how the various ECMs, stiffness, and multiple signaling regulate the fates of hypertrophic chondrocytes under the 3D condition, deepening our understanding of the mechanism behind the YAP signaling pathway. We suggest that combining in vivo cartilage models and further exploring how exactly YAP signaling acts on the activity of the ECM and physical properties may be used in new ways to treat obesity-related osteoarthritic diseases.

## Figures and Tables

**Table 1:** RT-PCR primers.

Target	Forward Primer (3′–5′)	Reverse Primer (5′–3′)
COL1A1	CGTGACCAAAAACCAAAAGT	GGGGTGGAGAAAGGAACAGA
COL2A1	GAGTGGAAGAGCGGAGACTACTG	CTCCATGTTGCAGAAGACTTTCA
COL10A1	GATCATGGAGCTCACGGAAAA	CCGTTCGATTCCGCATTG
SOX9	CTGAAGGGCTACGACTGCAC	TACTGGTCTCCCAGCTTCCT
GAPDH	TGAACGGGAAGCTCACTGG	TCCACCACCCTGTTCCGTA

## Data Availability

The datasets generated and analyzed during this study are available from the corresponding author upon reasonable request.

## Supplementary Materials

The following supporting information can be downloaded at: https://www.mdpi.com/article/10.3390/cells14161291/s1, Figure S1. Substrate stiffness influences focal adhesion formation in chondrocytes. Figure S2. Full-length uncropped Western blots corresponding to Figure 4 and validation of protein expression patterns by independent repeat experiments. Figure S3. Full-length uncropped Western blots corresponding to Figure 5 and validation of protein expression patterns by independent repeat experiments. Figure S4. Full-length uncropped Western blots corresponding to Figure 6 and validation of protein expression patterns by independent repeat experiments. Figure S5. Full-length uncropped Western blots corresponding to Figure 7 and validation of protein expression patterns by independent repeat experiments. Table S1. List of antibodies.
