# Supplementary material for: Substrate Stiffness Modulates Hypertrophic Chondrocyte Reversion and Chondrogenic Phenotype Restoration
Source: Cells. 2025 Aug 20;14(16):1291. doi: 10.3390/cells14161291 (PMC12385123; doi:10.3390/cells14161291)
Supplement: Supplementary file 1 [file cells-14-01291-s001.zip › cells-3719966-supplementary.pdf]

cells

MDPI

Article

Substrate Stiffness Modulates Hypertrophic Chondrocyte Reversion and Chondrogenic Phenotype Restoration

Da-Long Dong [1,2] and Guang-Zhen Jin [1,3,*]

[1] Nstitute of Tissue Regeneration Engineering (ITREN), Dankook University, Cheonan 31116, Republic of Korea; dongdalong@dankook.ac.kr
[2] Department of Nanobiomedical Science and BK21 PLUS NBM Global Research, Center for Regenerative Medicine, Dankook University, Cheonan 31116, Republic of Korea
[3] Department of Biomaterials Science, College of Dentistry, Dankook University, Cheonan 31116, Republic of Korea
* Correspondence: gzhjin2002@dankook.ac.kr; Tel.: +82-41-550-3082-4

Table S1. List of antibodies.

Antibody	Company	Item Number	Attributes	Dilution Ratio
Col2A1	Santa Cruz	sc-52658	Mouse	1：500
Col1A1	Cell Signaling	91144	Rabbit	1：1000
YAP (D8H1X)	Cell Signaling	14074S	Rabbit	1：1000
P-YAP	Cell Signaling	4911	Rabbit	1：1000
RUNX2	Santa Cruz	sc-390351	Mouse	1：500
β-actin	MeRCK	A3854	Mouse	1：20000
Smad1/5/8	Santa Cruz	sc-6031-R	Rabbit	1：500
Smad2/3 (C-8)	Santa Cruz	sc-133098	Mouse	1：500
Integrin β1/ITGB1	Santa Cruz	sc-13590	Mouse	1：500
FAK(D-1)	Santa Cruz	sc-271126	Mouse	1:300
Vinculin	abcam	129002	Rabbit	1:500
Paxillin(B-2)	Santa Cruz	sc-365379	Mouse	1:500
MMP13	abcam	39012	Rabbit	1:500

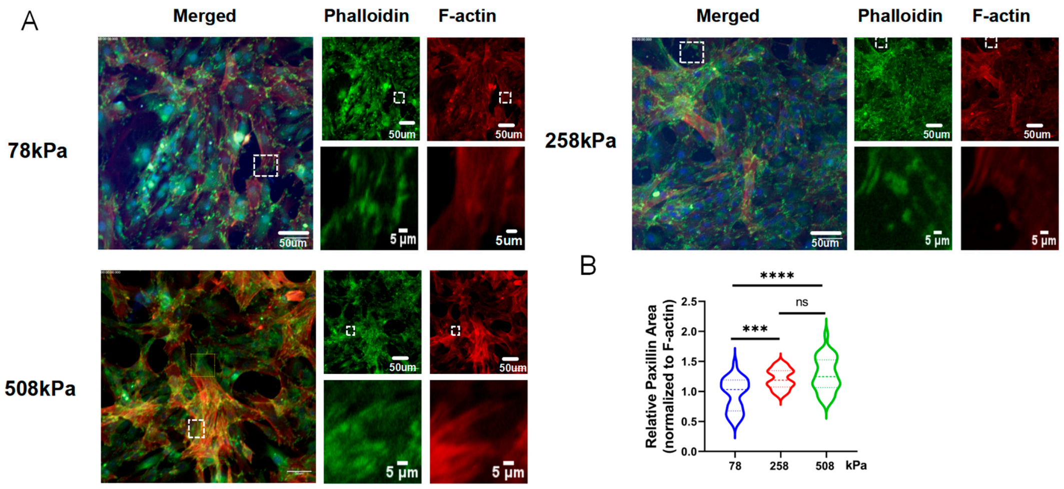

Figure S1. Substrate stiffness influences focal adhesion formation in chondrocytes. (A) Immunofluorescence staining of Paxillin (red) and F-actin (green, labeled with Phalloidin) in chondrocytes cultured on substrates of varying stiffness (78, 258, and 508kPa). Merged images and individual fluorescence channels are shown. Insets highlight enlarged views (scale bars: 5um) of representative focal adhesions. Paxillin signals are more prominent and densely clustered on stiffer substrates (508kPa), indicating enhanced focal adhesion formation. (B) Quantification of Paxillin-positive area normalized to F-actin signal across different stiffness groups. Data are presented as violin plots (mean±SD). Statistical analysis was performed using one-way ANOVA with post hoc test. ***$p < 0.001$; ****$p < 0.0001$; ns, not significant.

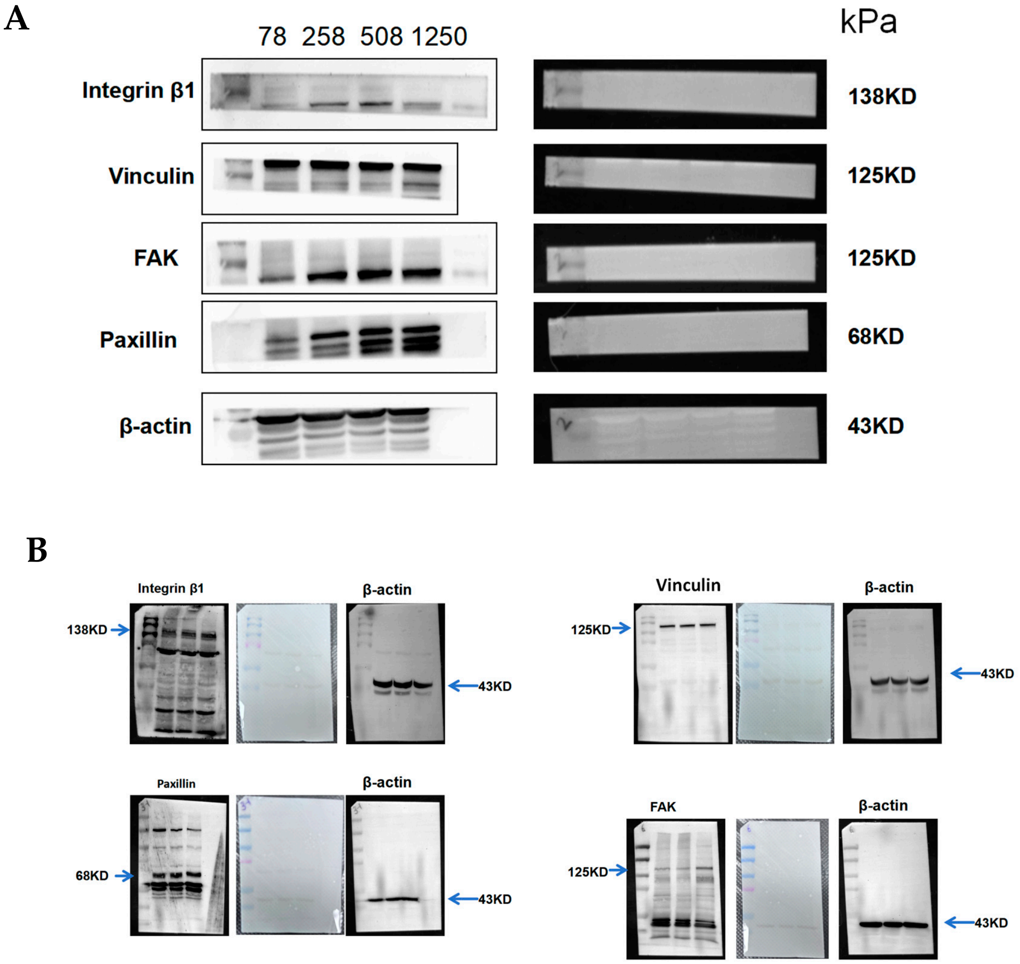

Figure S2. Full-length uncropped Western blots corresponding to Figure 4 and validation of protein expression patterns by independent repeat experiments. (A) Western blot analysis of Integrin β1, Vinculin, FAK, Paxillin, and β-actin in hypertrophic chondrocytes cultured on PDMS substrates with stiffnesses of 78, 258, and 508 kPa, as presented in the main manuscript (Figure 4). (B) Full-length uncropped Western blot images obtained from an independent repeat experiment conducted under the same conditions as those in the manuscript. The results are consistent with those in panel A, confirming the reproducibility and reliability of the findings. Molecular weights (kDa) are indicated on the left.

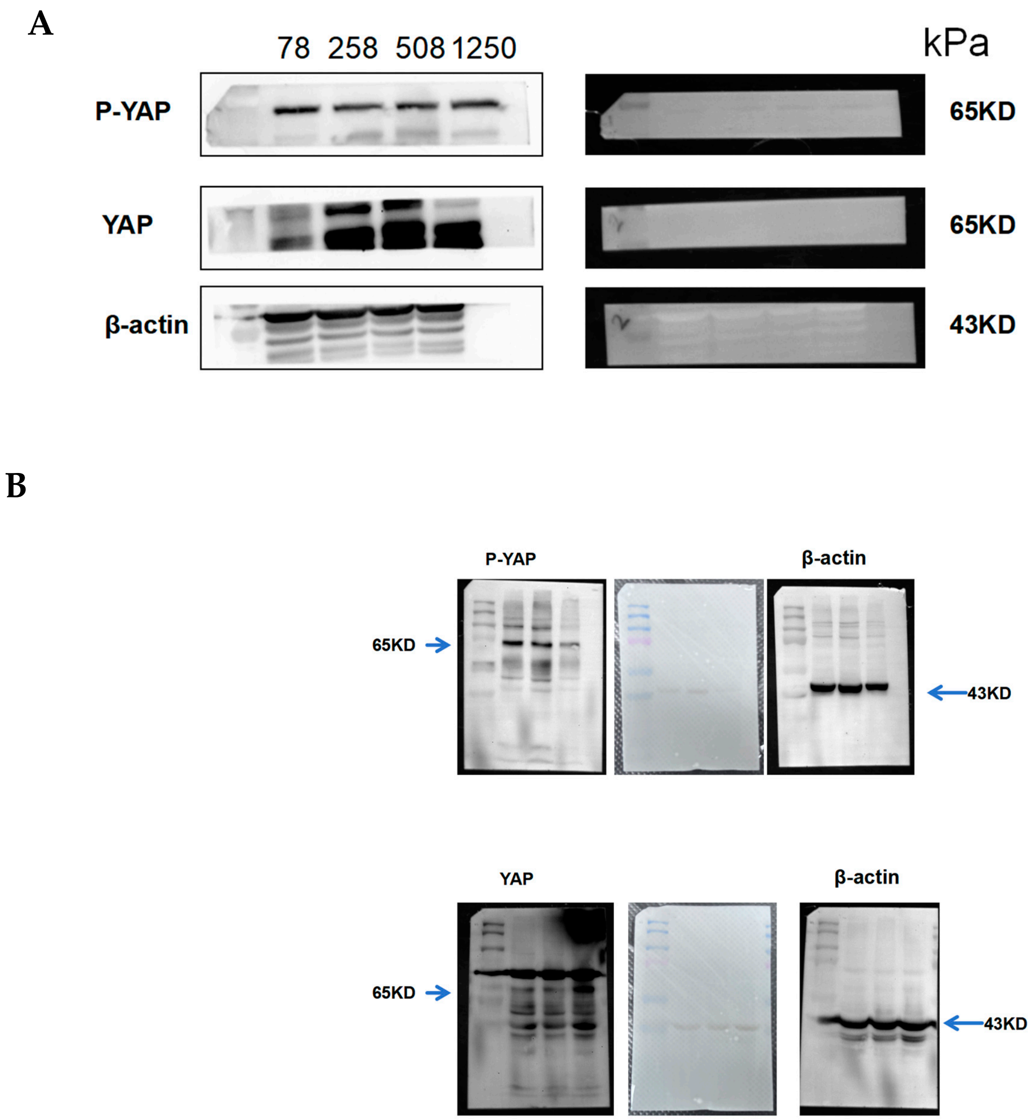

Figure S3. Full-length uncropped Western blots corresponding to Figure 5 and validation of protein expression patterns by independent repeat experiments. (A) Western blot analysis of YAP,P-YAP in hypertrophic chondrocytes cultured on PDMS substrates with stiffnesses of 78, 258, and 508 kPa, as presented in the main manuscript (Figure 5). (B) Full-length uncropped Western blot images obtained from an independent repeat experiment conducted under the same conditions as those in the manuscript. The results are consistent with those in panel A, confirming the reproducibility and reliability of the findings. Molecular weights (kDa) are indicated on the left.

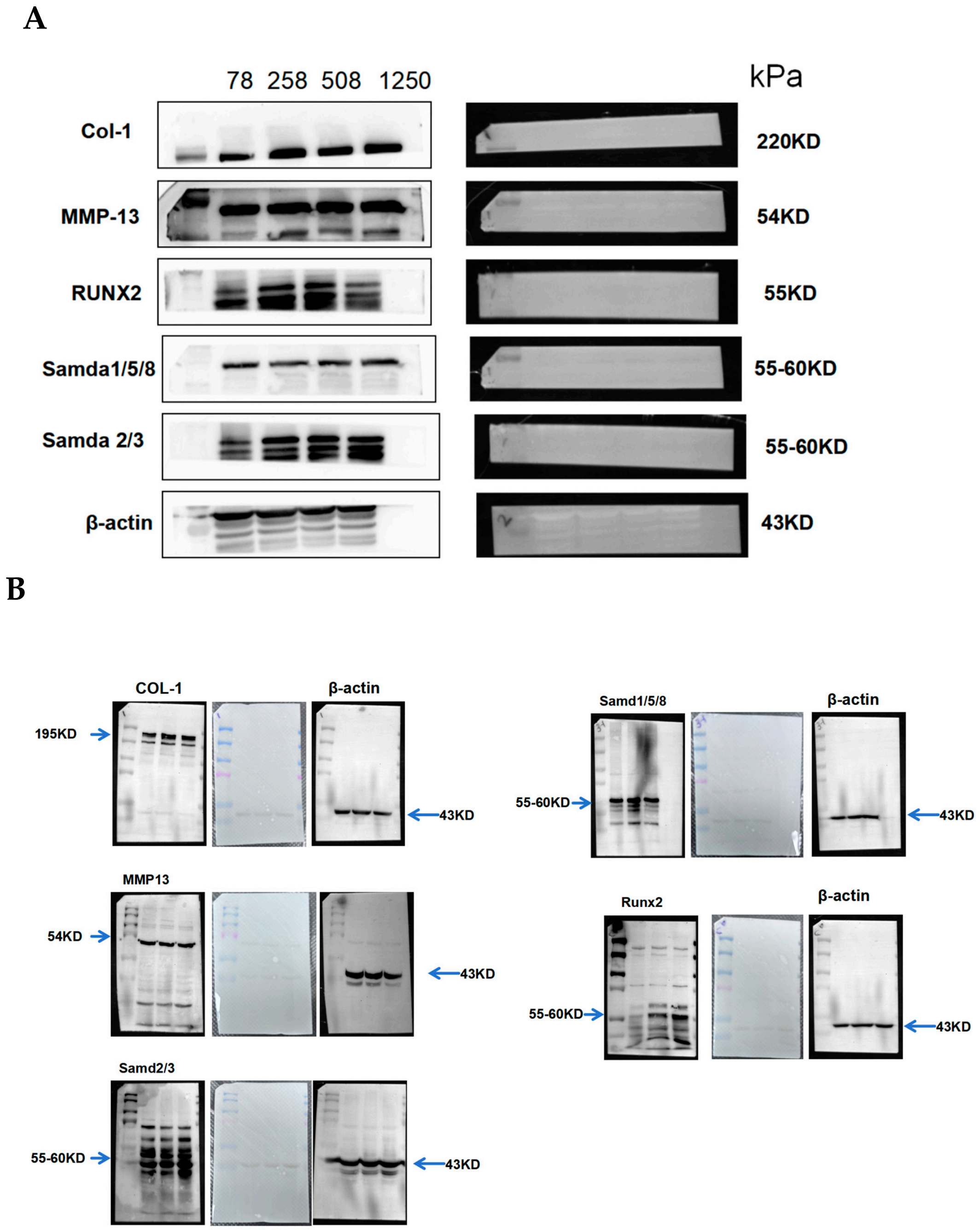

Figure S4. Full-length uncropped Western blots corresponding to Figure 6 and validation of protein expression patterns by independent repeat experiments. (A) Western blot analysis of Col-1，MMP-13,RUNX2,Samad1/5/8,Samad 2/3 in hypertrophic chondrocytes cultured on PDMS substrates with stiffnesses of 78, 258, and 508 kPa, as presented in the main manuscript (Figure 6). (B) Full-length uncropped Western blot images obtained from an independent repeat experiment conducted under the same conditions as those in the manuscript. The results are consistent with those in panel A, confirming the reproducibility and reliability of the findings. Molecular weights (kDa) are indicated on the left.

A

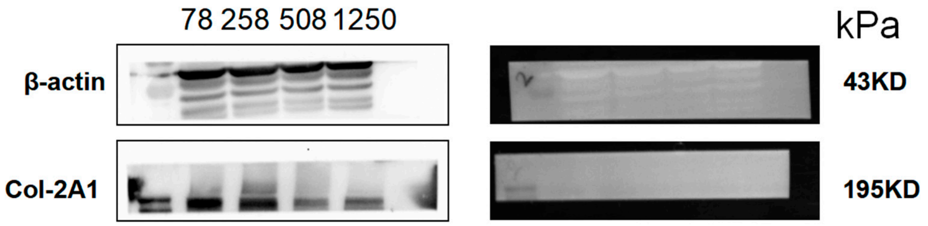

B

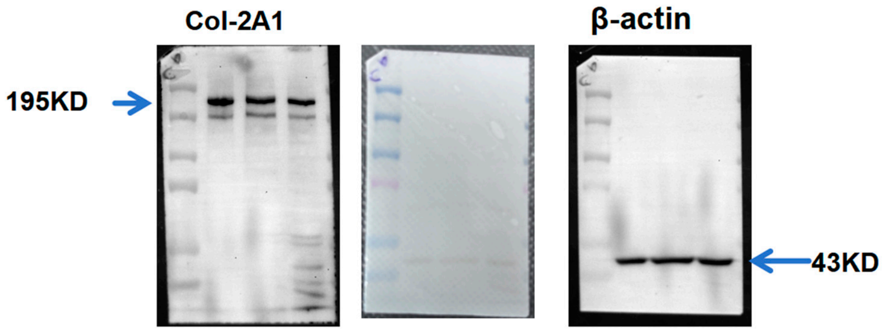

Figure S5. Full-length uncropped Western blots corresponding to Figure 7 and validation of protein expression patterns by independent repeat experiments.(A) Western blot analysis of Col-2A1 in hypertrophic chondrocytes cultured on PDMS substrates with stiffnesses of 78, 258, and 508 kPa, as presented in the main manuscript (Figure 7).(B) Full-length uncropped Western blot images obtained from an independent repeat experiment conducted under the same conditions as those in the manuscript. The results are consistent with those in panel A, confirming the reproducibility and reliability of the findings. Molecular weights (kDa) are indicated on the left.
